# Deep learning in GPCR drug discovery: benchmarking the path to accurate peptide binding

**DOI:** 10.1093/bib/bbaf186

**Published:** 2025-04-26

**Authors:** Luuk R Hoegen Dijkhof, Teemu K E Rönkkö, Hans C von Vegesack, Jacob Lenzing, Alexander S Hauser

**Affiliations:** Department of Drug Design and Pharmacology, University of Copenhagen, Jagtvej 160, 2100 Ø, Copenhagen, Denmark; Center for Pharmaceutical Data Science, University of Copenhagen, Denmark; Department of Drug Design and Pharmacology, University of Copenhagen, Jagtvej 160, 2100 Ø, Copenhagen, Denmark; Center for Pharmaceutical Data Science, University of Copenhagen, Denmark; Department of Drug Design and Pharmacology, University of Copenhagen, Jagtvej 160, 2100 Ø, Copenhagen, Denmark; Center for Pharmaceutical Data Science, University of Copenhagen, Denmark; Department of Drug Design and Pharmacology, University of Copenhagen, Jagtvej 160, 2100 Ø, Copenhagen, Denmark; Center for Pharmaceutical Data Science, University of Copenhagen, Denmark; Department of Drug Design and Pharmacology, University of Copenhagen, Jagtvej 160, 2100 Ø, Copenhagen, Denmark; Center for Pharmaceutical Data Science, University of Copenhagen, Denmark

**Keywords:** GPCR, AlphaFold, RoseTTAFold, peptide receptor, structure prediction, docking

## Abstract

Deep learning (DL) methods have drastically advanced structure-based drug discovery by directly predicting protein structures from sequences. Recently, these methods have become increasingly accurate in predicting complexes formed by multiple protein chains. We evaluated these advancements to predict and accurately model the largest receptor family and its cognate peptide hormones. We benchmarked DL tools, including AlphaFold 2.3 (AF2), AlphaFold 3 (AF3), Chai-1, NeuralPLexer, RoseTTAFold-AllAtom, Peptriever, ESMFold, and D-SCRIPT, to predict interactions between G protein-coupled receptors (GPCRs) and their endogenous peptide ligands. Our results showed that structure-aware models outperformed language models in peptide binding classification, with the top-performing model achieving an area under the curve of 0.86 on a benchmark set of 124 ligands and 1240 decoys. Rescoring predicted structures on local interactions further improved the principal ligand discovery among decoy peptides, whereas DL-based approaches did not. We explored a competitive tournament approach for modeling multiple peptides simultaneously on a single GPCR, which accelerates the performance but reduces true-positive recovery. When evaluating the binding poses of 67 recent complexes, AF2 reproduced the correct binding modes in nearly all cases (94%), surpassing those of both AF3 and Chai-1. Confidence scores correlate with structural binding mode accuracy, which provides a guide for interpreting interface predictions. These results demonstrated that DL models can reliably rediscover peptide binders, aid peptide drug discovery, and guide the selection of optimal tools for GPCR-targeted therapies. To this end, we provided a practical guide for selecting the best models for specific applications and an independent benchmarking set for future model evaluation.

## Introduction

Protein–protein interactions (PPIs) drive essential biological processes, including signaling pathways, gene regulation, and structural assembly. Although PPIs can be experimentally determined, limited throughput has driven the development of deep learning (DL) methods to predict protein complex structures from sequences. The accuracy of these models is evaluated in the Critical Assessment of Structure Prediction (CASP) [1] experiment, a community-wide initiative to benchmark structural modeling tools for unpublished experimental structures. In the CASP15 experiment [1, 2] (2022), all the top-performing models were based on DL, akin to AlphaFold 2 (AF2) [3]. Recent advances in PPI modeling have improved the accuracy from 8% in CASP14 (2020) to nearly 50% in CASP15 [4].

Although CASP experiments comprehensively evaluate the prediction accuracy for both monomeric and multimeric protein targets, the performance of modeling tools in specific applications differs. Of particular interest are their potential use as screening methods to identify novel ligands for G protein-coupled receptors (GPCRs), which play a pivotal role in cellular signaling by transmitting extracellular signals to intracellular responses. Owing to their central role in (patho)physiology, GPCRs are the largest drug-target family with unexplored targets and disease applications [5, 6]. Peptides represent a critical class of GPCR ligands that regulate numerous physiological processes including neurotransmission, metabolism, and immune responses. Thus, identifying novel peptides computationally holds great therapeutic potential, particularly for orphan GPCRs for which endogenous ligands are still unknown or poorly characterized [7, 8]. However, predicting GPCR–peptide interactions poses unique challenges compared with general PPI predictions. Challenges include limited structural data, dynamic conformational complexity, and the highly diverse nature of peptide ligand sequences, lengths, and modifications. Hence, a detailed assessment of DL tools for GPCR–peptide modeling is required to accelerate drug discovery.

To address this gap, we assessed several state-of-the-art PPI prediction tools for their ability to classify peptide binders as true binders or non-interacting peptides. These tools include two types of models: (1) large language models (LLMs) that estimate the probability of a positive interaction without predicting the structure of the GPCR–peptide complex and (2) structural modeling tools that provide predictions of full GPCR–peptide complexes alongside innate confidence metrics of predicted interactions. We used innate confidence metrics alongside alternative energy-based, DL-pretrained, and calculated scoring metrics to optimize a classifier to accurately distinguish cognate binders from decoys. Additionally, we tested a competitive tournament approach, in which multiple peptides simultaneously compete for the GPCR-binding pocket. To determine whether structural accuracy drives the classification performance of structure-aware DL, we benchmarked their performance on a recent set of experimental GPCR–peptide complexes that have not been used to train the respective models.

## Results

### Structural models accurately distinguish endogenous ligands from non-binders

To comprehensively assess the classification accuracy of the selected DL tools, we curated a set of all GPCRs with endogenous principal peptide ligands (Supplementary Fig. 1). The dataset encompasses a diverse representation of receptor types, including 105 class A, 15 class B1, and 3 class F GPCRs. To emulate the high incidence of negative peptide interactions in real-world peptide screening, we selected 10 non-binding human endogenous peptides as decoys for each cognate GPCR–ligand pair tested to be rediscovered by the respective approach.

**Figure 1:**
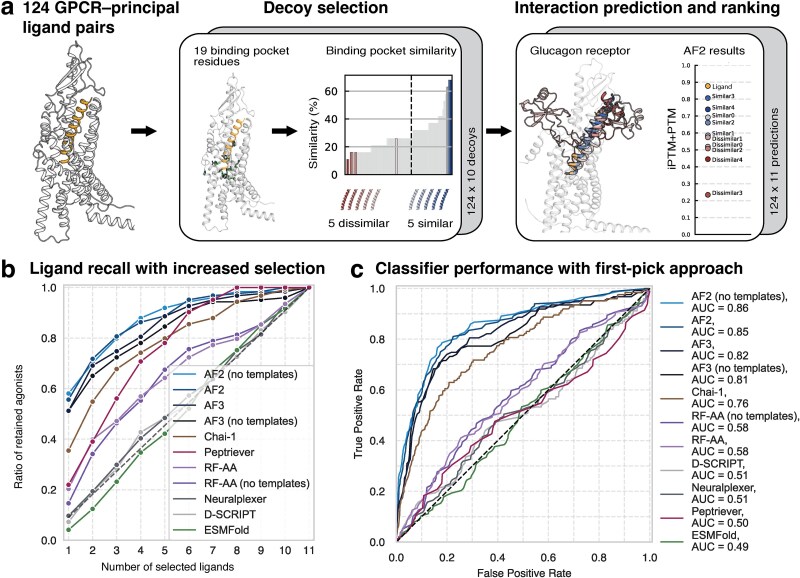
Benchmarking classifier performance. (a) A benchmarking dataset was generated consisting of GPCR–peptide pairs, where decoys were selected based on binding pocket similarity between binding pocket generic residues for each decoy and GPCR endogenous peptide (orange) pair. The dataset includes 124 GPCR–principal ligand and 1240 GPCR–decoy pairs. The glucagon receptor is shown as an example, with 11 AF2 complex predictions superimposed. The ipTM + pTM score ranks the ligand (glucagon) first and separates it from the decoys. (b) We evaluated ligand recall when incrementally selecting more peptides from the ranked set of 11 peptides for each GPCR. AF2 (without templates) performs best, ranking the principal ligand first among 10 decoys for 58% of GPCRs. (c) Model performance as classifiers, evaluating 124 binders and 1240 decoys. Only the highest-ranked ligand among the 11 per receptor is considered positive. Supplementary Table 3 shows extended performance metrics for only similar and only dissimilar decoys.

Decoy peptides were selected from endogenous ligands, with dissimilar decoys from distant GPCRs with 20% pocket similarity and the most similar from receptors with 62% pocket similarity (Supplementary Fig. 2). The resulting dataset consisted of 124 principal ligand–GPCR pairs and 1240 decoy pairs (Fig. 1a). Employing a 10:1 decoy-to-binder ratio balances realistic screening conditions across peptide GPCRs, with computational tractability for different DL tools and conditions. By challenging the model’s capacity to distinguish true peptide binders from closely related non-binding decoys, the benchmark poses a stringent classification challenge and provides a fair comparison for applications in peptide discovery, peptide selectivity, and structure–activity relationships. This independently constructed and publicly available benchmark dataset of GPCR−peptide decoys may guide future efforts to evaluate molecular docking performance, similar to past benchmark datasets [9].

We benchmarked eight tools to produce 13 640 unique GPCR–peptide interaction predictions. We first evaluated scalable LLMs with fast inference times, including Peptriever [10] and D-SCRIPT [11]. Although Peptriever performed strongly with increased ligand selection, its initial recall was low when only the top-ranked ligand was retained (Fig. 1b). This low initial recall corresponds to its poor classifier performance in Receiver operating characteristic (ROC) curve analysis, which considers only a balanced dataset of one positive and one negative peptide per GPCR (Fig. 1c). D-SCRIPT failed to show a better-than-random performance in the classifier task (Supplementary Table 1).

Next, we investigated structural models, including AF2, AF3, Chai-1, RF-AA, NeuralPLexer, and ESMFold. RF-AA distinguished ligands from decoys but was outperformed by other structural models (Fig. 1c). Chai-1 attained an area under the curve (AUC) of 0.76 but was outperformed by both AF3 (AUC = 0.82) and top-performing AF2 (AUC = 0.86). For instance, for the glucagon receptor, the AF2 ipTM + pTM metric successfully ranked the principal ligand (glucagon) first, while similar decoys were ranked higher than dissimilar decoys, as expected, owing to greater binding pocket similarity (Fig. 1a). We tested the ranking performance across all 124 GPCRs, and the performance of AF2 was consistent with this example. When considering ligand recall, AF2 (without templates) outperformed the other models, ranking the principal ligand first among the decoys for 58% of GPCRs (Fig. 1b). In addition, AF2 demonstrated superior performance when evaluated using a first-pick classifier approach against the most dissimilar decoy, with comparable results to those of AF3 (Fig. 1c).

Structural templates improved AF3 classifier performance (Supplementary Table 2), but not for AF2 or RF-AA (Wilcoxon signed-rank test, *P* = .482 and *P* = .309, respectively). Other models, including ESMFold and NeuralPLexer, failed to enrich peptide binders from decoys (Supplementary Table 3). These results underscore the strong discriminatory power of multiple sequence alignments (MSAs) in peptide binding prediction and ranking.

Given AF2’s superior classification accuracy, we also tested its ability to place the principal ligand in the orthosteric binding pocket when GPCRs were simultaneously modeled with multiple peptides. The binding pocket was defined by eight GPCR backbone positions mainly located in the transmembrane domains (Supplementary Fig. 3). Simultaneous peptide modeling significantly accelerated predictions (Supplementary Fig. 4 and Supplementary Table 4); however, the ability of AF2 to correctly place the principal ligand in the orthosteric binding pocket dropped quickly once more decoy peptides were included in the model (Supplementary Fig. 5, more detail in Supplementary Results and Methods).

### Rescoring improves classifier performance

**Figure 2:**
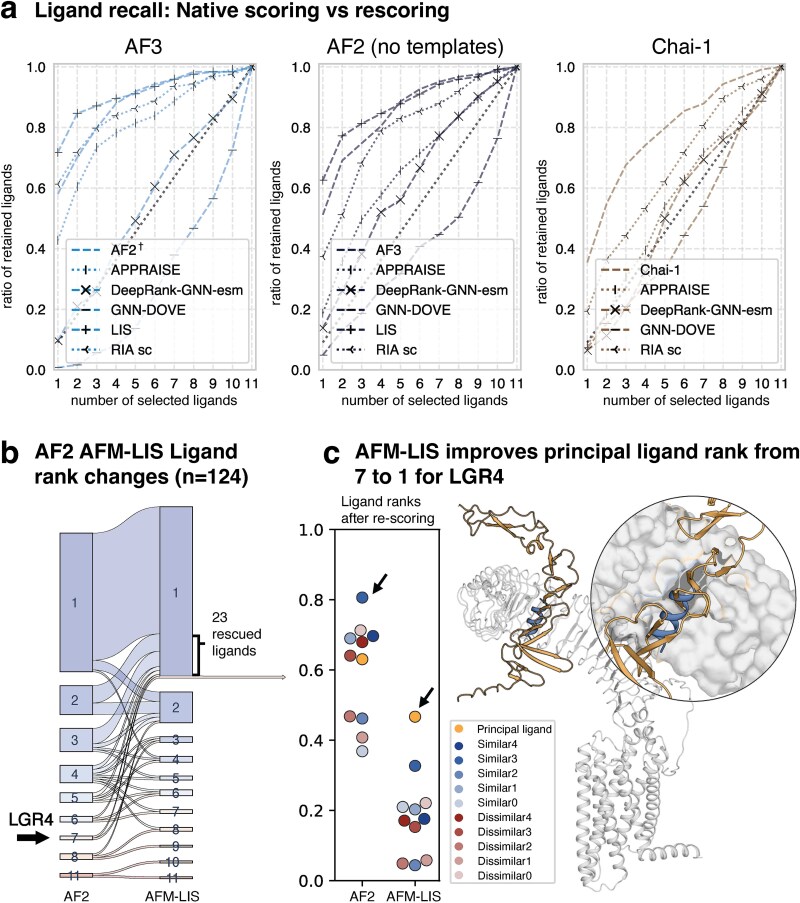
Classifier rescoring. (a) The cognate ligand retention of the best base models and the effect of rescoring on performance. The retention at random class assignment is shown as a dashed line. AF2 without templates (^†^), AF3 (without templates), and Chai-1 were included as the best-performing base models. (b) The ranking comparison of AF2 and AFM-LIS for 124 principal ligands. After rescoring AF2 complexes, AFM-LIS correctly reassigns 23 misranked ligands to rank 1 but misranks 6 previously correct ligands. Overall, the improvement in ranking performance is statistically significant (Wilcoxon signed-rank test, *P* = .0018). (c) The largest improvement in rank after AFM-LIS rescoring was for lgr4. The default AF2 scoring metric ipTM + pTM (before) and AFM-LIS (after) are shown for the peptides, with principal ligand R-spondin-4 (GtP ID: 3700) moving from rank 7 to rank 1. The predicted complex is displayed, and the shorter decoy that was initially ranked first is superimposed onto the structure.

Structural accuracy is crucial for determining the binding strength of GPCR–peptide complexes, as the shape of the binding pocket dictates ligand compatibility. Structural rescoring tools aim to refine model predictions by reinterpreting the structural models [12–14]. RoseTTAInterfaceAnalyzer (RIA) and APPRAISE score physical attributes of predicted structures, while AFM-LIS refines the predicted aligned error (PAE) of local interactions from the AF output for its confidence metric [13]. DL-based methods, such as GNN-DOVE [15] and DeepRank-GNN-esm [16], are trained to rank candidate models based on learned interactions. To benchmark the rescoring methods for GPCR–peptide classification, we applied energy-, confidence-, and DL-based rescoring methods to all 1364 complexes predicted by AF3, AF2, Chai-1, and RF-AA. Most rescoring methods did not improve the performance (Fig. 2a). For AF3, RF-AA, and Chai-1, the shape complementarity (SC) metric ranked highest among the RIA metrics (Supplementary Fig. 6) but did not surpass the baseline performance (Fig. 2a).

Interestingly, AFM-LIS significantly improved the ligand ranking (*P* = .0018, Wilcoxon signed-rank test). For both AF2 and AF3, single-model AFM-LIS runs showed performance comparable to that of the default five-model setup (*P* = .156 and *P* = .751, respectively), suggesting a runtime-efficient option for some use cases (Supplementary Table 5). For AF2, AFM-LIS primarily improved the high-ranking ligands, with >50% of those previously ranked second or third, achieving the best rank after AFM-LIS rescoring (Fig. 2b). AFM-LIS notably improved the ranking of GPCR LGR4 (Fig. 2c), elevating the principal ligand from the seventh to the first position.

### Peptide length, binding affinity, and GPCR selectivity influence classifier performance

**Figure 3:**
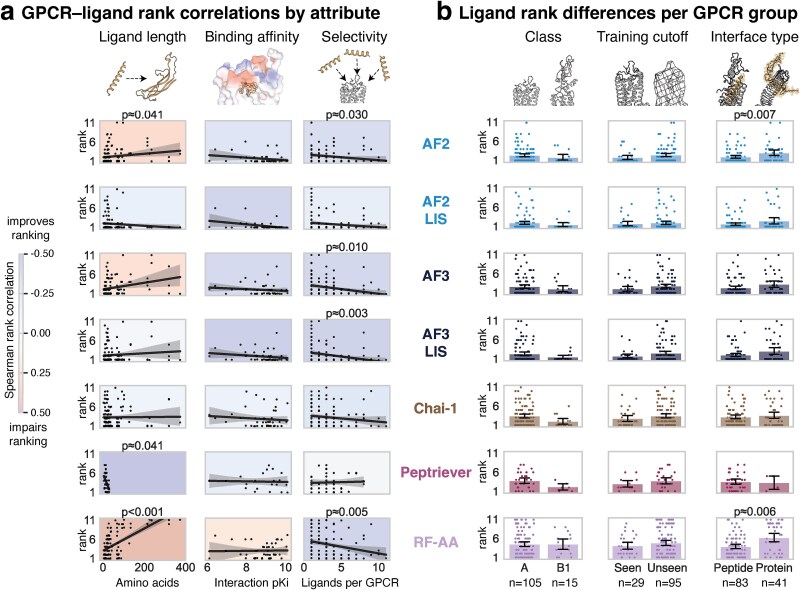
Classifier sub-analysis. (a) Spearman’s rank correlations between complex attributes and ranking performance, with per-attribute Bonferroni-corrected *P*-values shown for significant correlations. Positive correlations indicate that ligands are ranked worse as the attribute increases, while negative correlations show that increasing the attribute improves rank performance. (b) A comparison of ligand rankings for different GPCR classifications, including class, presence of resolved complexes in training data (seen), and interface type based on receptor family. Significance is indicated by Wilcoxon signed-rank tests, with per-attribute Bonferroni-corrected *P*-values shown if *P* <.05.

To explore the factors influencing GPCR–peptide ranking in the best-performing models, we conducted a sub-analysis of GPCR and peptide attributes across 124 GPCR–peptide pairs (Fig. 3a). The N-terminal length of the GPCRs marginally affected ranking (*P* > .05) (Supplementary Table 6). The number of endogenous peptides per GPCR ranged from 1 to 11 (Supplementary Fig. 7). GPCRs with a higher number of ligands, such as those in the neuropeptide or chemokine receptor families, demonstrated better ranking performance than less promiscuous GPCRs. Conversely, the number of GPCR targets per peptide ranged from one to seven, with no correlation with ranking (Supplementary Table 6). Among the 45 interactions with binding affinities (pKi), higher-affinity interactions exhibited a non-significant trend toward improved ranking (Fig. 3a).

Peptide length was inversely correlated with AF2 and RF-AA rankings, favoring shorter ligands (Fig. 3a). Unexpectedly, RF-AA ranked ligands with deeper MSAs worse, likely because of the difficulty with longer peptides. In contrast, Peptriever performed better on longer ligands, with its performance peaking at its 30-residue length limit. For both AF2 and AF3, AFM-LIS rescoring mitigated the negative impact of peptide length on the ranking. GPCRs with pretraining structures (“seen” complexes) were ranked slightly better across all models, but the Bonferroni correction rendered the trend insignificant (*P* = 1.00) (Fig. 3b, Supplementary Table 7). Chai-1 and Peptriever ranked class B1 GPCRs higher than class A GPCRs, a trend that was not observed in MSA-based methods (Fig. 3b). The MSA-based models ranked GPCRs with explicit peptide-binding pockets more favorably than those with protein interfaces, with a significant effect on AF2 (*P* = .01) and RF-AA (*P* = .01) (Fig. 3b). This preference was not observed in the embedding-based models Peptriever and Chai-1.

### AF2 outperforms AF3, Chai-1, and RF-AA on GPCR–peptide structural accuracy

**Figure 4:**
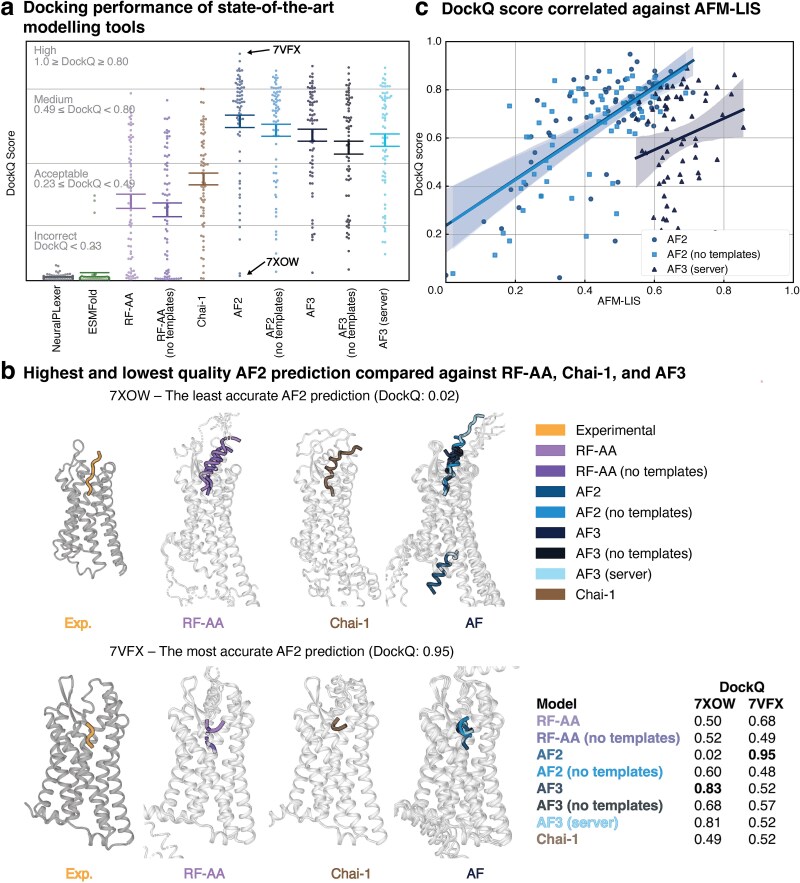
Structural benchmark of GPCR–peptide interactions published after the training date cutoff for all included modeling tools. (a) DockQ score distributions of 67 predicted GPCR–peptide pairs for each model. AF2 (with and without structural templates) and AF3 outperform all other included models. (b) Performance of Chai-1, AF, and RF-AA on two sample structures from the structural benchmark set, according to the DockQ scores of AF2 predictions ranging from worst (0.02, PDB code, 7XOW) to best (0.95, PDB code, 7VFX). (c) AFM-LIS and DockQ scores displayed a significant Spearman’s rank correlation for AF2 predictions with and without templates (*r*(65) = .67, *P* < .0001, *r*(65) = .67, *P* < .0001), while this correlation was not significant for AF3 (server) predictions (*r*(65) = .20, *P* = .097).

We assessed how the best-performing classification models were performed in terms of structural accuracy. To allow for an unbiased approach, we included 67 experimental GPCR–peptide models published after the training date cutoff (30 September 2021) for the included models (Supplementary Fig. 8). This dataset covered 47 unique receptors and 58 peptide ligands. We used the DockQ score [17] to quantify the structural accuracy of docking (Fig. 4a). The DockQ score ranges from 0 to 1, and predictions are considered acceptable when the DockQ score is ≥0.23 [17]. We used the root mean squared deviation (RMSD) between all the GPCR backbone α-carbons of the predicted and experimental models to measure the accuracy of the predicted GPCR conformations (Supplementary Fig. 9). Pairwise comparisons of DockQ and RMSD for each model using the paired Wilcoxon signed-rank test are provided in Supplementary Tables 8.1 and 8.2, respectively, with the most important results highlighted.

AF2 failed to reproduce acceptable docking in only four (templates) and five (without templates) cases (Fig. 4a). Structural templates significantly improved the predictive quality of AF2 (*P* < .001) but not AF3 (*P* = .164). Surprisingly, AF3 (with templates) and the AF3 server failed to reproduce the binding modes in six models, whereas AF3 (without templates) failed in eight inputs. AF2 (with templates) produced significantly more accurate binding modes than all AF3 configurations (*P* < .01). However, the template-free models for AF2 were not significantly more accurate than those generated by AF3 (with templates, *P* = .84; without templates, *P* = .084; and AF3 server, *P* = .265). There was no significant difference in the prediction accuracy of the local installation of AF3 and the webserver (*P* = .28).

The weakest-performing models, NeuralPLexer and ESMFold (mean DockQ: 0.015 and 0.025, respectively), failed to produce acceptable predictions in nearly all cases. NeuralPLexer failed to reproduce all 67 GPCR–peptide interfaces and the predicted GPCR backbone α-carbons deviated 5.09 Å on average while the average RMSD for other tools ranged from 0.92 Å (AF2) to 1.98 Å (RF-AA, with templates). Although ESMFold only produced acceptable docking for three inputs, the average GPCR α-carbon backbone accuracy of the predictions (1.55 Å) was significantly better than that generated by RF-AA without structural templates (1.81 Å) (*P* < .01).

RF-AA failed to predict the interactions in 26 and 28 of the 67 models (with and without templates, respectively). In 11 cases, RF-AA folded the receptor sequence into its binding pocket (Supplementary Fig. 10a) and inaccurate predictions were produced for seven inputs where the GPCR had large extracellular domains (Supplementary Fig. 10b). Notably, Chai-1 significantly outperformed RF-AA (*P* < .05) but did not yield as high an accuracy as AF2 (*P* < .0001) or AF3 (*P* < .0001), with or without templates.

The visualized models (Fig. 4b) highlight the predictions of the best-performing models in two instances based on the AF2 (with templates) prediction quality. The most accurate AF2 prediction (PDB: 7VFX, DockQ: 0.95, receptor RMSD: 0.54 Å) was based on fMet-Leu-Phe receptor (FPR1) in complex with peptide ligand fMet-Ile-Phe-Leu. Chai-1, RF-AA, AF2 (without templates), and AF3 showed acceptable docking of the same interaction, although RF-AA (with templates) placed the peptide ~9.9 Å deeper into the binding pocket than the experimental model. The worst AF2 prediction was obtained for the Cholecystokinin-2 receptor (GASR) in complex with gastrin-17 (PDB: 7XOW; DockQ: 0.02; receptor RMSD: 0.54 Å). AF2 (with templates) placed the peptide on the intracellular side of the receptor, whereas RF-AA, Chai-1, and AF3 docked it correctly. Interestingly, when AF2 was run without templates for the same input, the prediction was more accurate (DockQ, 0.60; receptor RMSD: 1.07 Å).

To determine whether confident predictions were produced robustly, the top-performing models, Chai-1, AF2, and AF3, were run with five unique seeds, and the additional predictions were scored using the DockQ score. The results showed that, although the average standard deviation of the DockQ scores for AF2 was the lowest among the three models, the DockQ score was not significantly affected by the random seeds in any of the models (full results from the Friedman test are provided in Supplementary Table 9).

When considering the average time required to generate structural predictions, AF2 was the slowest as generating a single prediction took 144.2 s (with templates), while a single prediction generated by AF3 required 17.8 s (with templates). Although Chai-1 was significantly slower than AF3 (*P* < .0001), Chai-1 was not run using MSAs, while RF-AA, AF2, and AF3 prediction times reported here excluded the MSA preparation time (Supplementary Fig. 11 and Supplementary Table 10).

### GPCR–peptide pairing performance correlates with structural accuracy

To investigate the relationship between structural accuracy and classification performance, we correlated the AFM-LIS with the DockQ score (Fig. 4c). The DockQ score positively correlated with AFM-LIS for template-free (*r*(65) = .67, *P* < .0001) and default AF2 models (*r*(65) = .67, *P* < .0001), but not for AF3 (server) (*r*(65) = .20, *P* = .097).

We also explored whether the structural accuracy of the predicted GPCR–peptide complexes was driven by the presence of training structures with high sequence identity (see Supplementary Methods). RF-AA, AF2, and AF3 all generalized well to novel inputs with low training sequence identities, as the DockQ score was not significantly correlated with the closest training sequence identity in RF-AA, AF2, and AF3 at *P* < .05 when analyzed using Spearman’s rank correlation (Supplementary Fig. 12).

## Discussion

**Figure 5:**
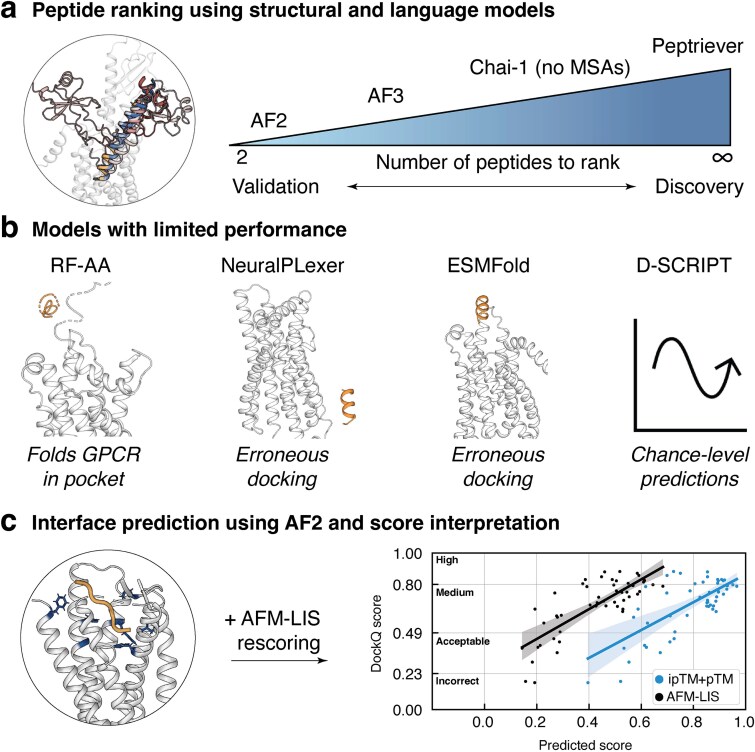
Recommended workflow for GPCR–peptide binding prediction. (a) Based on the results from the classifier benchmark, AF2, AF3, Chai-1, and Peptriever perform best in rediscovering principal ligands among decoys. These four models are recommended for different use cases as the structure-aware models require more computational resources compared to LLM-based methods. AF3 and Chai-1 (without input MSAs) offer viable alternatives to AF2, given their faster runtimes while delivering medium to acceptable DockQ-quality interfaces. Even though LLM-based Peptriever shows low initial recall, it can be applied at much larger scale for initial enrichment of positive interactions. (b) We caution users about RF-AA’s tendency to misfold GPCRs into the binding pocket. NeuralPLexer and ESMFold fail to dock peptides correctly, and D-SCRIPT predictions remain at chance level for GPCR–peptide interactions. (c) Based on the presented data, AF2 is recommended for accurate GPCR–peptide interface predictions. We provide an overview for interpreting confidence metrics (ipTM + pTM and AFM-LIS) for expected docking quality (DockQ score).

Previous studies have benchmarked the accuracy of structural modeling tools for GPCRs and other membrane proteins [18–20], including insights into AF2 and AF3 [21, 22]. We expanded these efforts by simultaneously benchmarking a diverse set of models for peptide-binder classification and structural accuracy on an independent benchmark set from known endogenous interactions. GPCR peptide ligands exhibit diverse binding profiles, which require models to capture both explicit transmembrane pockets and broader protein interfaces for accurate predictions. Structure-based tools outperform sequence-based models for PPI prediction, with AF2 being the best modeling tool for GPCR–peptide interactions, both in terms of classification and structural accuracy. We summarized these findings by providing a recommended GPCR–peptide prediction workflow for either specific case validation or high-throughput discovery approaches, including considerations for interface prediction, peptide scoring, and model limitations (Fig. 5).

Models with high structural accuracy, particularly AF2 and AF3, outperformed the others in true ligand identification, showing that structural accuracy is strongly correlated with interaction prediction. The inclusion of structural templates sometimes biased the intracellular docking of the peptide, likely due to the availability of GPCR–G protein templates for these complexes. Competitive modeling accelerates predictions but decreases true-positive recovery, limiting its use in large-scale screening.

Interestingly, AF3 did not yield more structurally accurate predictions than AF2 did. According to AF3 authors, the most notable performance improvements were observed in antibody–protein interaction modeling [23]. These gains likely do not apply to the included GPCR–peptide interfaces, which have more cross-entity evolutionary data than antibody–protein interfaces. Because AF3 generated predictions significantly faster than AF2 with no notable difference in the structural accuracy of template-free predictions, AF3 is preferable for large-scale screening owing to its small tradeoff between accuracy and runtime.

AFM-LIS [13] is the only rescoring metric that enhances classification performance. It is significantly correlated with the DockQ score [17], implying that AFM-LIS extracts confidence from relevant local interactions, even with disordered interface regions. RIA’s SC metric performed similarly to the native AF2 and RF-AA scores, highlighting its potential utility for structural models lacking reliable confidence metrics. Beyond the scoring metrics studied here, pDockQ2 similarly extracts information from AF2’s native scoring metrics to best correlate the structural confidence of the predicted interfaces with the true docking performance [20].

RF-AA’s poor performance may be due to two factors. First, its native scoring metric, PAE-inter, applies only to nucleic acids or small-molecule interfaces. Second, RF-AA accurately modeled the GPCR transmembrane regions but often misfolded extracellular regions. The transmembrane regions of GPCRs are often most confidently modeled using cryo-EM and X-ray crystallography, while intracellular and extracellular loops are not always expressed in the constructs. Therefore, it is possible that the training data aids RF-AA in accurately modeling these regions. Further studies should investigate why such models are produced using RF-AA and not AF2 or AF3, although all models use similar modeling strategies and training data.

Among the best-performing models, we analyzed their dependence on various peptide and GPCR attributes for the classification performance. AF2 ranked shorter peptides and ligands for highly promiscuous GPCRs better, implying that these binding sites are more adaptable or inherently flexible, enabling them to adopt conformations that readily accommodate various related peptides. This observation aligns with findings from a study reporting higher success rates for delimited peptides in receptor docking [24] and with findings that shorter sequences tend to exhibit higher mean pLDDT scores [25]. In terms of classification, the models showed a trend of improved ranking for class B1 over class A GPCRs (Fig. 3d), which likely resulted from the complete structural coverage of class B1 receptors [26].

DL models, such as D-SCRIPT and NeuralPLexer, may suffer inflated performance owing to reliance on sequence similarity, interaction counts, or signal peptide biases [27, 28]. Based on NeuralPLexer’s training description, it was only trained to model homomeric PPIs, aiming to model homomeric receptor complexes with small-molecule ligands [29] and potentially explaining the poor quality of GPCR–peptide predictions. Its hierarchical diffusion-based approach is optimized for atomic-level accuracy [29], which may limit its ability to capture the large-scale conformational rearrangements necessary for peptide-induced receptor binding. Peptriever performed well despite being an LLM, suggesting effective prevention of data leakage using training partitions based on gene-based groupings [10]. Although Peptriever can be effective for overall enrichment, its low initial recall limits its utility for identifying true peptide binders. While structural models achieved high classifier performance with a decoy-to-ligand ratio of 10:1, performance expectations should be adjusted for real-world screening, where negative interactions are more prevalent and hit rates typically fall in the low single-digit percentage range [30].

Ranking performance did not correlate significantly with MSA depth, although previous studies have shown that AF2 performance can be improved by querying larger biological sequence databases to build deeper MSAs [31]. To circumvent the computational cost of deep MSAs, LLM-based protein embeddings may be used to model co-evolutionary relationships [32]. For example, Chai-1 and ESMFold used embeddings from the ESM-2 protein language model instead of MSAs [33]. The poor structural and classification performance of ESMFold likely stems from its lack of fine-tuning for PPIs, with the architecture’s three recycling steps possibly being insufficient for flexible interfaces [34]. Its reliance on single-sequence embeddings without evolutionary constraints could further limit its ability to capture conserved interaction patterns, and the absence of diffusion-based sampling might reduce its ability to model peptide-induced receptor conformational changes. However, Chai-1 generated more structurally accurate predictions than RF-AA and its runtime was significantly faster than that of AF2 (Supplementary Table 10). The results from MSA-free modeling using Chai-1 are particularly promising because MSA-based methods have previously outperformed LLM-based methods in terms of structural accuracy [33].

New models such as RF2-ppi [35] and Boltz-1 [36] will continue to emerge, highlighting the importance of gold standard benchmarking practices. Recent advancements in protein structure prediction have been extended to model a broader range of biomolecular entities, including amino acids, small molecules, ions, DNA, and RNA [23, 29, 37]. Future studies should assess whether these tools improve ligand docking and evaluate their ability to model post-translational modifications, conformational states, genetic variability, and larger structural complexes, such as dimers, G proteins, and arrestins [38–40].

## Methods

### Classifier benchmark

To evaluate the performance of the computational models for GPCR–peptide pairing, we constructed a dataset of human GPCRs and their respective peptide ligands. These interactions were obtained using the Pygtop API (version 2.1.3) from the IUPHAR/BPS Guide to Pharmacology [41] (GtP) (https://pygtop.readthedocs.io/en/latest/). Selection was limited to endogenous peptides in humans, classified as agonists or full agonists, with uniquely annotated sequences. The full dataset contained 124 GPCRs identified by their GPCRdb [42] (https://gpcrdb.org/) ID and 207 peptides identified by their sequence. The principal ligand was selected for each GPCR. Of all the GPCRs, 32 had only one ligand and 38 mentioned the principal ligand(s) in the GtP comment field. The shortest peptide was selected based on the identification of multiple principal ligands. For the remaining 54 GPCRs, the shortest peptide with the experimental activity was chosen as the principal ligand. For the eight GPCRs with missing experimental data for the human receptor, the shortest principal ligand with the measured experimental activity in mice was chosen. This resulted in a dataset of 410 unique GPCR–peptide interactions, of which 124 were principal interactions, forming the basis for the classifier benchmark dataset. In 22 of the 124 GPCRs, the principal ligand was shared by multiple receptors and two of the principal ligands were paired with five GPCRs. As some of the included modeling tools did not allow for unnatural amino acids represented by wildcard characters (X) in the input sequences, the unnatural amino acids were replaced with their closest matching natural amino acids.

The benchmark dataset was designed with a principal ligand-to-decoy ratio of 1:10 to represent the rarity of the binding ligands during peptide screening. Pairwise BLOSUM62 binding pocket similarities of all 124 GPCRs were calculated using a set of generic residues from classes A, B, and F GPCRs. For each receptor, 10 principal ligands of other GPCRs were selected as decoys. Two types of decoys were used: five dissimilar and five similar. Five dissimilar decoys with <35% sequence similarity were randomly selected from all the principal GPCR ligands (https://gpcrdb.org/services/alignment/similarity/). Similar decoys were selected on the basis of the principal ligands of the top five GPCRs with the highest binding pocket similarity. The GPCRs with shared principal ligands were excluded from the search for similar decoys. This classifier dataset with 1364 GPCR–peptide pairs is inherently difficult to solve because models must be trained to learn the real binding mode and cannot rely solely on differences in topology.

We used eight ML-based methods to predict the interaction probability of 1364 GPCR–peptide complexes: ESMFold [34], AF2 [3], AF3 [23], RF-AA [37], Peptriever [10], D-SCRIPT [11], Chai-1 [33], and NeuralPLexer [29]. AF2 (version 2.3.1) was run using the multimer preset with default settings, both with and without templates, except that the number of generated predictions was reduced to five from the default 25. UniRef90 [43] (Dec 2023), BFD (Mar 2019), and MGnify [44] (Feb 2023) were used to build the MSAs using the default tools described in the original publication. Template-free modeling was enabled by setting the maximum template date cutoff to 1 January 1950. AF3 (version 3.0.0) was run using the default settings, both with and without templates. UniRef90 [43] (Nov 2024), BFD (Mar 2019), and MGnify [44] (Mar 2024) were used to build MSAs using the default tools described in the original publication [23]. The PDB70 database [45] (version 22-03-19) and RCSB PDB [46] (PDB files retrieved in Jan 2025) were used to search for structural templates for both AF2 and AF3 predictions.

Similar to AF2 and AF3, RF-AA was run with and without templates. Both receptor and peptide sequences were used as protein inputs to the model. For template-free modeling, the n_templ parameter was set to zero, whereas this parameter was maintained at the default value (n_templ = 4) for all other predictions. The templates for RF-AA were searched from the PDB100 (Mar 2021) database supplied by the authors, while the MSAs were built using UniRef30 [43] (version 2020_06) and BFD (Mar 2019) databases, as in the original publication. ESMFold models were generated using the esmfold_v1 pretrained model weights provided at https://github.com/facebookresearch/esm, with no additional changes to the default parameters recommended by the developers [34]. NeuralPLexer models were also generated using default inference parameters, and both peptide and receptor sequences were provided as inputs using the input receptor parameter. Chai-1 (version 0.5.2) was run locally without using the MSA server or structural templates, as previously described (https://github.com/chaidiscovery/chai-lab/tree/v0.5.2) [33].

Interaction scores between zero and one were predicted for 1364 GPCR–peptide pairs using several 2D and 3D predictive models. The selected metric from each model for scoring the GPCR–peptide pairs is shown in Supplementary Table 11. For ligand recall analysis, the predicted scores of the decoys and ligands were sorted for all 124 GPCRs. The percentage of retained ligands was calculated by sequentially selecting peptides, starting from the top-ranked peptide and continuing until all the 11 peptides were included. For ROC curve analysis, the top-ranked peptide for each GPCR was designated as positive, whereas the other peptides were labeled as negative. The ROC curve was plotted using Python Scikit-learn. This “first-pick” method was applied to data subsets when including all decoys, dissimilar decoys only, similar decoys only, and only the most dissimilar decoy.

The four top-performing models, AF2, AF3, Chai-1, and RF-AA, were evaluated for classifier performance when applying the rescoring methods. For AF2 and AF3, the predicted complex with the highest ipTM + pTM score was used for rescoring. For RF-AA, the complex with the lowest PAE was used, whereas for Chai-1, the highest-scoring complex was used. For all four models, rescoring was performed for RIA, DOVE-GNN, Deeprank-GNN-esm, and APPRAISE (single-peptide approach) version 1.2. Additionally, AFM-LIS rescoring was performed for AF2 (no template) and AF3. The metrics used per rescoring tool are listed in Supplementary Table 11. Ranking performance after rescoring was compared using the Wilcoxon signed-rank test using scipy.stats.

The effects of various GPCR and peptide attributes on their assigned ranks were assessed using Spearman’s rank correlation from scipy.stats. The predicted ranks of the 124 principal ligands, ranging from 1 to 11, were correlated with attribute values or categories. The Bonferroni *P*-value correction was applied per attribute.

The discrete and continuous attributes included peptide length, AF2 peptide MSA depth, and ligand selectivity (the number of GPCRs to which a peptide is known to bind) based on ligand interactions from GtP. Similarly, the GPCR selectivity is defined as the number of peptides that target a given GPCR. Additionally, the interaction pKi values for 45 ligands (39 class A and 6 class B GPCRs) were obtained from GtP. The nominal categories included GPCR class, GPCR interface, and whether the complex was seen during training as per the 30 September 2021 training date cutoff of AF3. The interfaces of the GPCRs were categorized as 83 and 41 peptide interfaces based on the ligand type categorization as per the GPCRdb.

### Structural benchmark

To evaluate the performance of the chosen structure prediction tools, a benchmark set of recently released GPCR–peptide structures was retrieved from GPCRdb (20 December 2024) [42]. A cutoff date after 30 September 2021 was used to ensure that the included structures were not used to train any of the models. As none of the included tools support unnatural amino acids as inputs, we only modeled peptide and receptor sequences with natural amino acids. Both peptide and receptor sequences were parsed from the SEQRES field of the PDB entries of the experimental structures so that the predictions were based on fully expressed amino acid sequences and not only structurally characterized residues. All PDB codes and other relevant information regarding the chosen experimental structures are listed in Supplementary Table 12. The accuracy of the predicted GPCR–peptide interactions was measured using the DockQ score [17] (https://github.com/bjornwallner/DockQ/).

As described previously, AF2 (version 2.3.1) was run using the multimer preset with default settings (with and without templates) to generate the default number of complex predictions. However, updated AF3 sequence databases were used to model the complexes in the structural benchmark. The AF3 server (https://golgi.sandbox.google.com/) was run using default settings, and AF3, RF-AA, ESMFold, NeuralPLexer, and Chai-1 were run using the same settings as described earlier. All models were run on NVIDIA A100 GPUs and the runtimes were compared using the Wilcoxon signed-rank test.

**Key Points**

- The largest independent benchmark of DL approaches for GPCR–peptide complex prediction and peptide rediscovery.
- Computational peptide screening was demonstrated as feasible using endogenous ligands and highly similar decoys.
- Rescoring on local interactions, but not DL or energy-based calculations, improves pairing predictions.
- AlphaFold2 correctly docked 94% of peptides, surpassing AlphaFold3 and Chai-1 on unseen experimental complexes.
- Overview of methods for accurate binding prediction and peptides, addressing limitations and use cases.

## Supplementary Material

Supplementary_Figures_bbaf186

Supplementary_Tables_bbaf186

Supplementary_Methods_bbaf186

Supplementary_Results_bbaf186

## Data Availability

All data supporting the findings of this study are provided in the Supplementary Tables. The benchmarking set and open-source code used to conduct the analyses is available at GitHub (https://github.com/HauserGroup/GPCR_peptide_benchmarking). The source code and supporting data are publicly available for download on Zenodo at https://zenodo.org/records/15014285.
