# Supplementary material for: Deep learning in GPCR drug discovery: benchmarking the path to accurate peptide binding
Source: Brief Bioinform. 2025 Apr 26;26(2):bbaf186. doi: 10.1093/bib/bbaf186 (PMC12031724; doi:10.1093/bib/bbaf186)
Supplement: Supplementary_Figures_bbaf186 [file supplementary_figures_bbaf186.docx]

**SUPPLEMENTARY FIGURES**

**
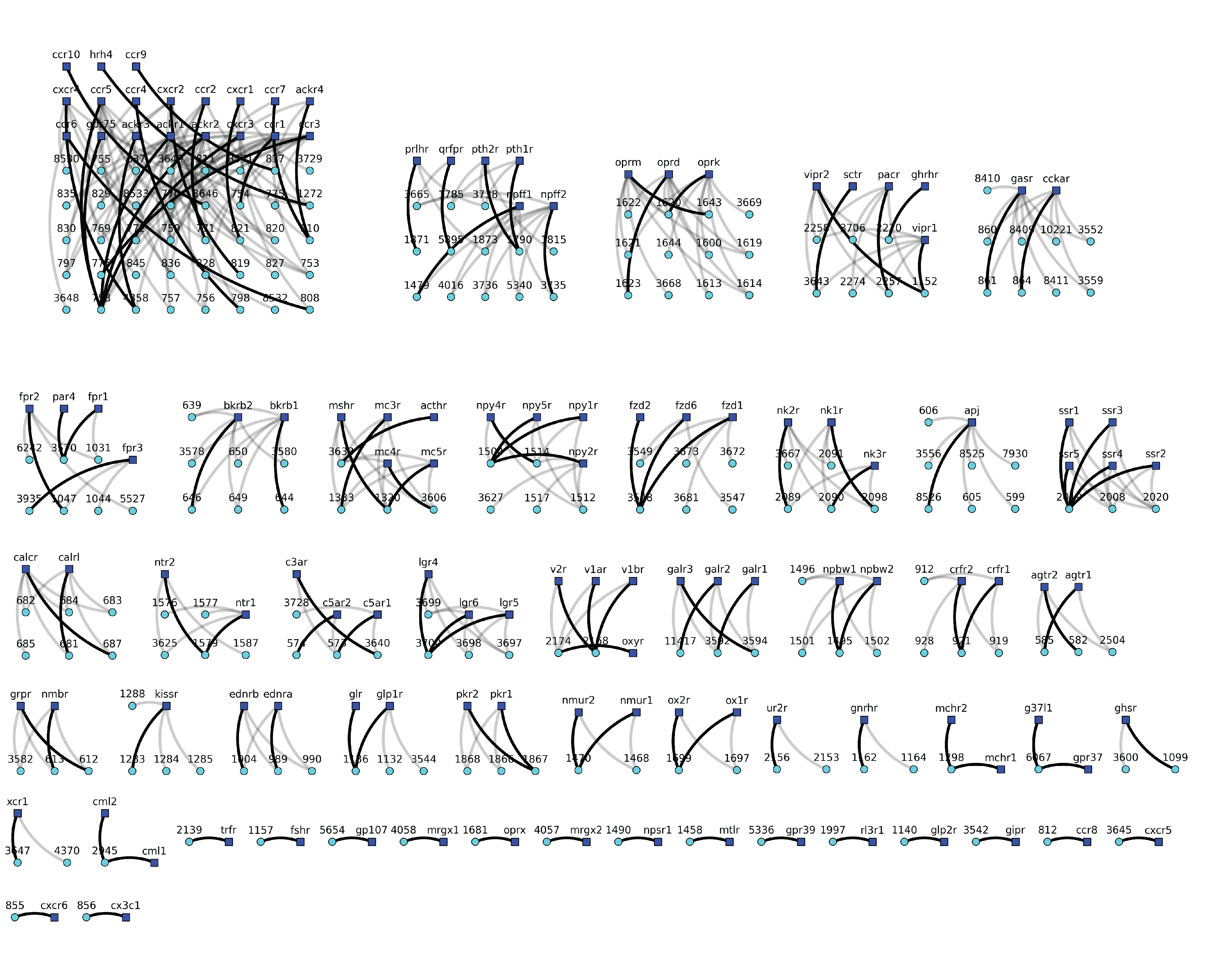
**

**Supplementary Figure 1: Network of GPCR-peptide interactions.** The 411 interactions obtained from the IUPHAR Guide to Pharmacology database are shown, with the chosen principal agonists highlighted as a black line. The 124 GPCRs are shown as dark blue squares and the peptides as cyan circles. From this network, the distribution of GPCR selectivity and agonist selectivity is obtained.


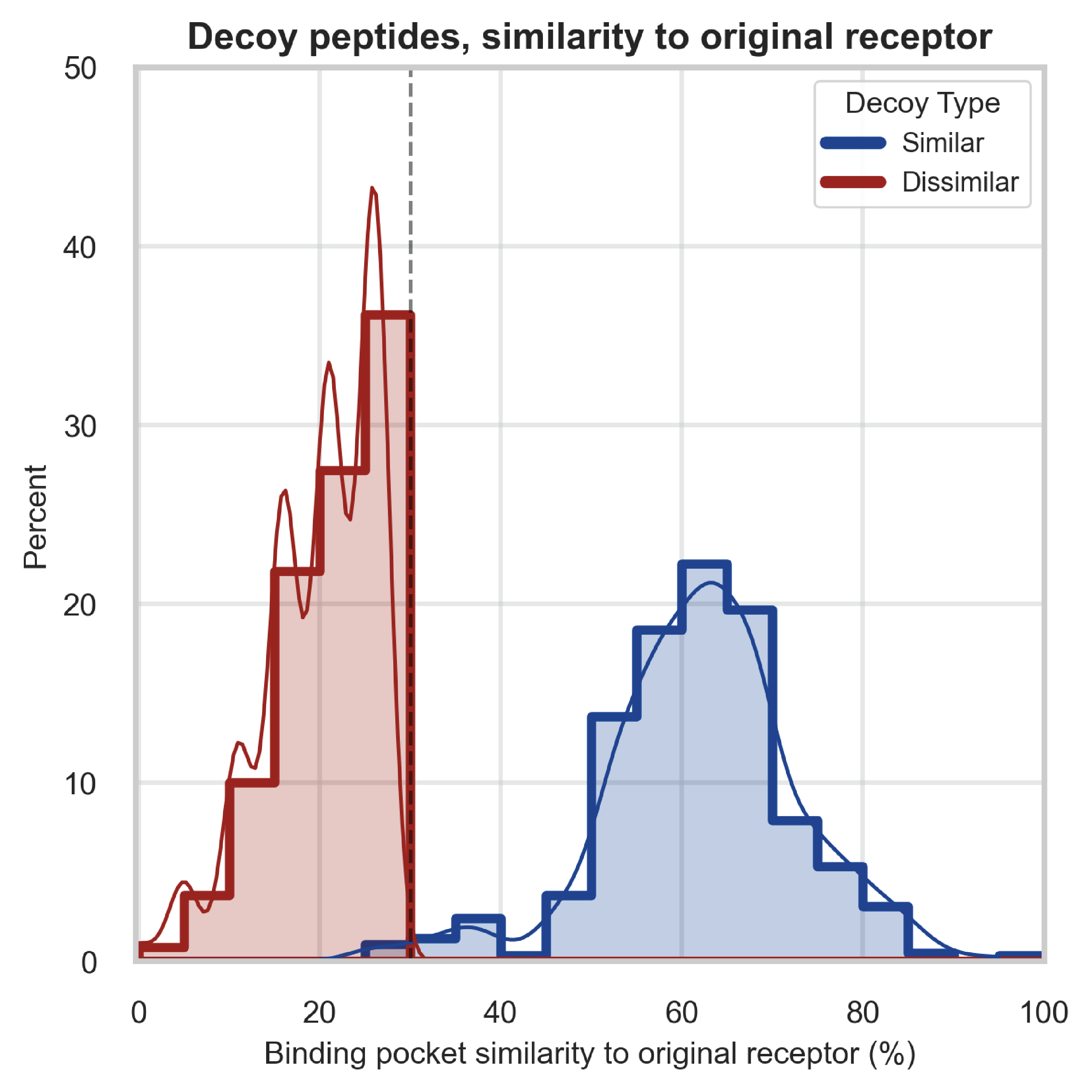


**Supplementary Figure 2: Decoy BLOSUM62 similarity distributions.** For the 1,240 decoys, the binding pocket similarity distribution is shown per decoy type. Dissimilar decoys average 19.97% binding pocket similarity, while similar decoys average 62.21% binding pocket similarity.


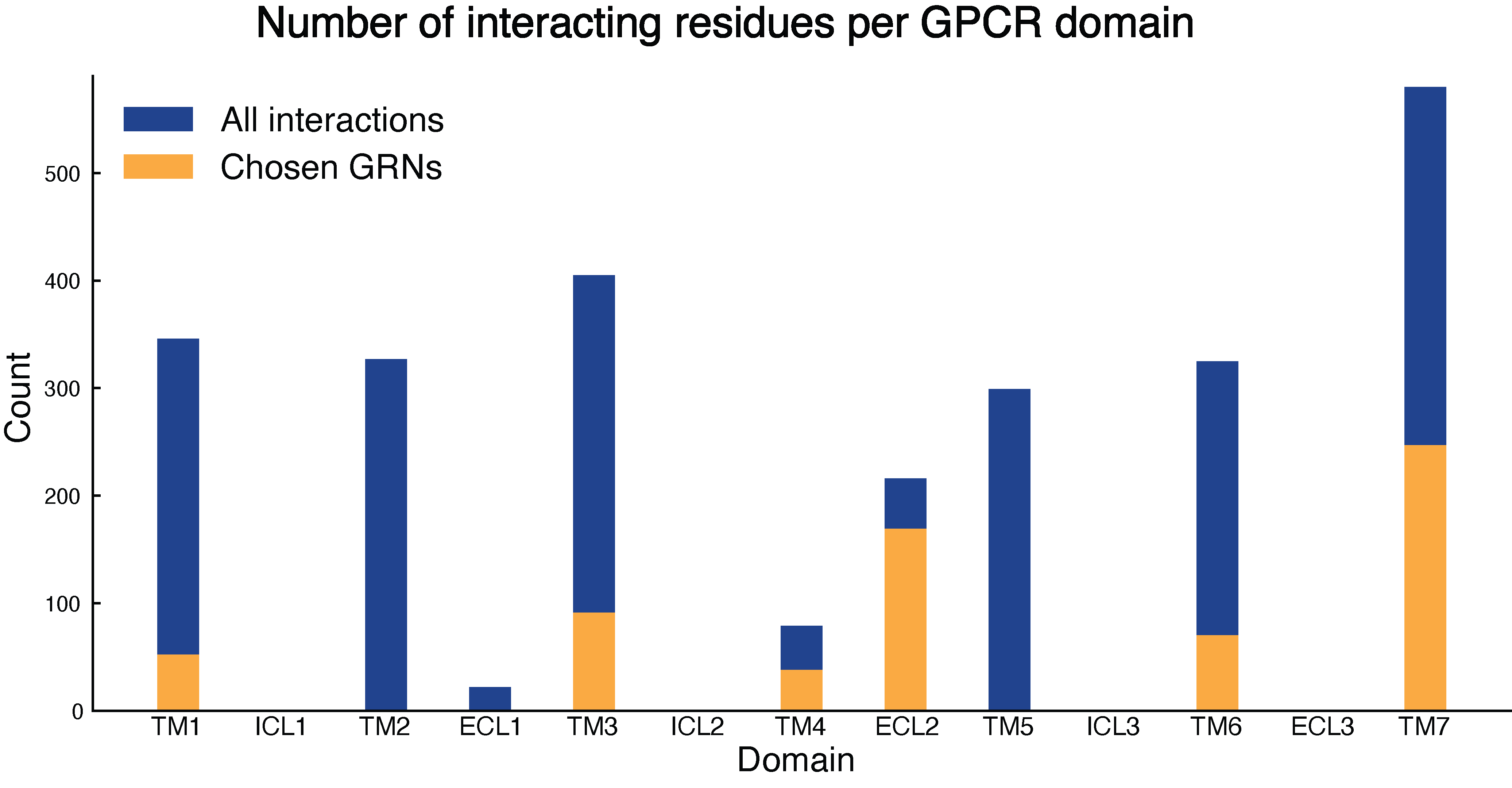


**Supplementary Figure 3: Distribution of GRNs involved in GPCR–peptide interactions across different GPCR domains.** Interaction GRNs across 148 experimental GPCR–peptide models. The most frequently observed GRNs are in TM3, TM7, and the 2^nd^ extracellular loop (ECL2).


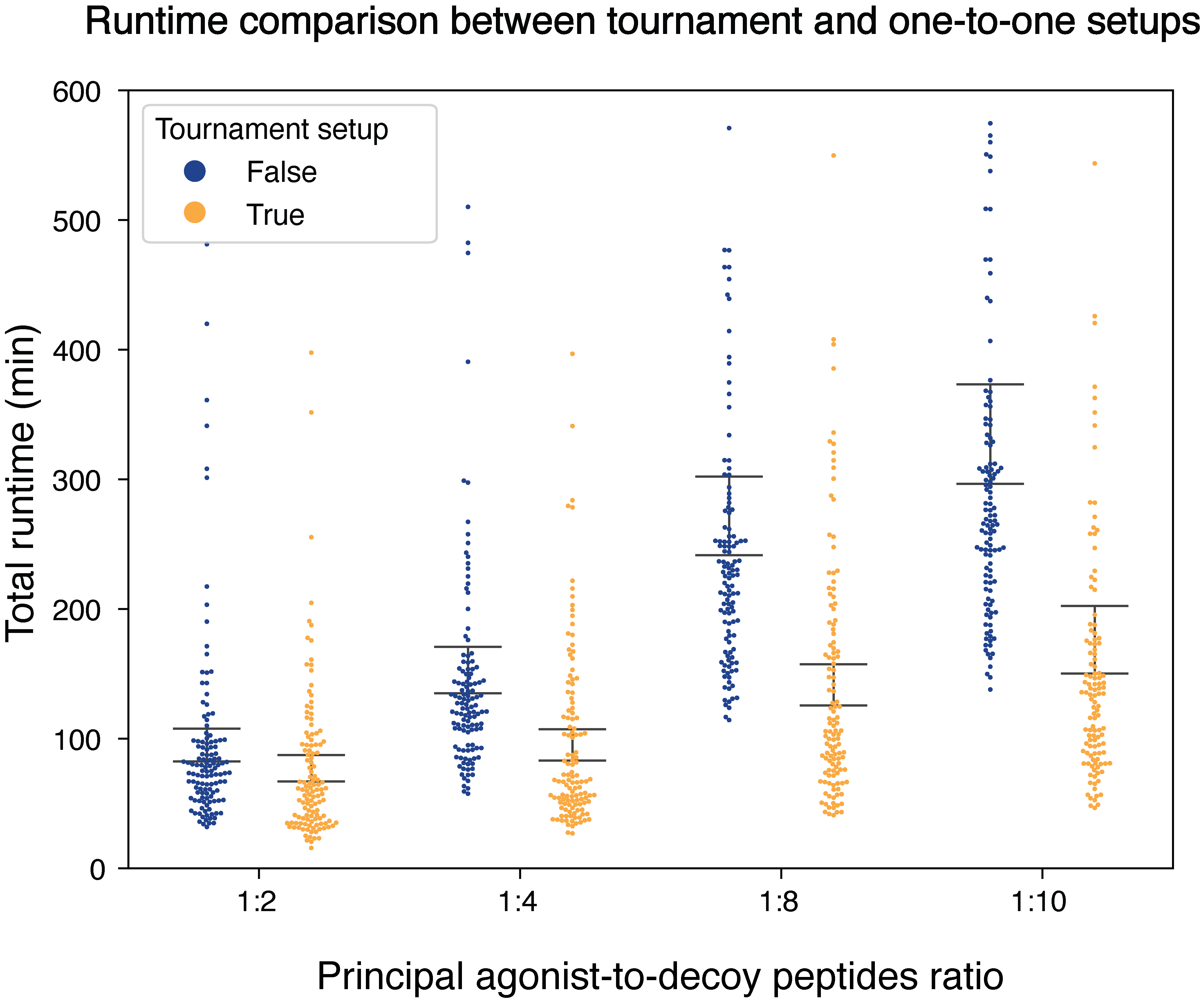


**Supplementary Figure 4: Runtime comparison between AF2 modelling using tournament setup against total runtime of the same interactions modelled in one-to-one fashion.** To see whether modelling GPCR targets with multiple peptides simultaneously (true) results in significantly faster modelling runtime, we compared the runtimes of AF2 complex predictions of GPCRs with increasing number of decoy peptides against the cumulative runtime of modelling the same interactions in one-to-one fashion (false), producing individual models for each GPCR–peptide interactions. The visualized runtime is an average of the five generated models by AF2. The figure shows that tournament setup results in significantly faster runtimes in principal agonist-to-decoy ratios.

**
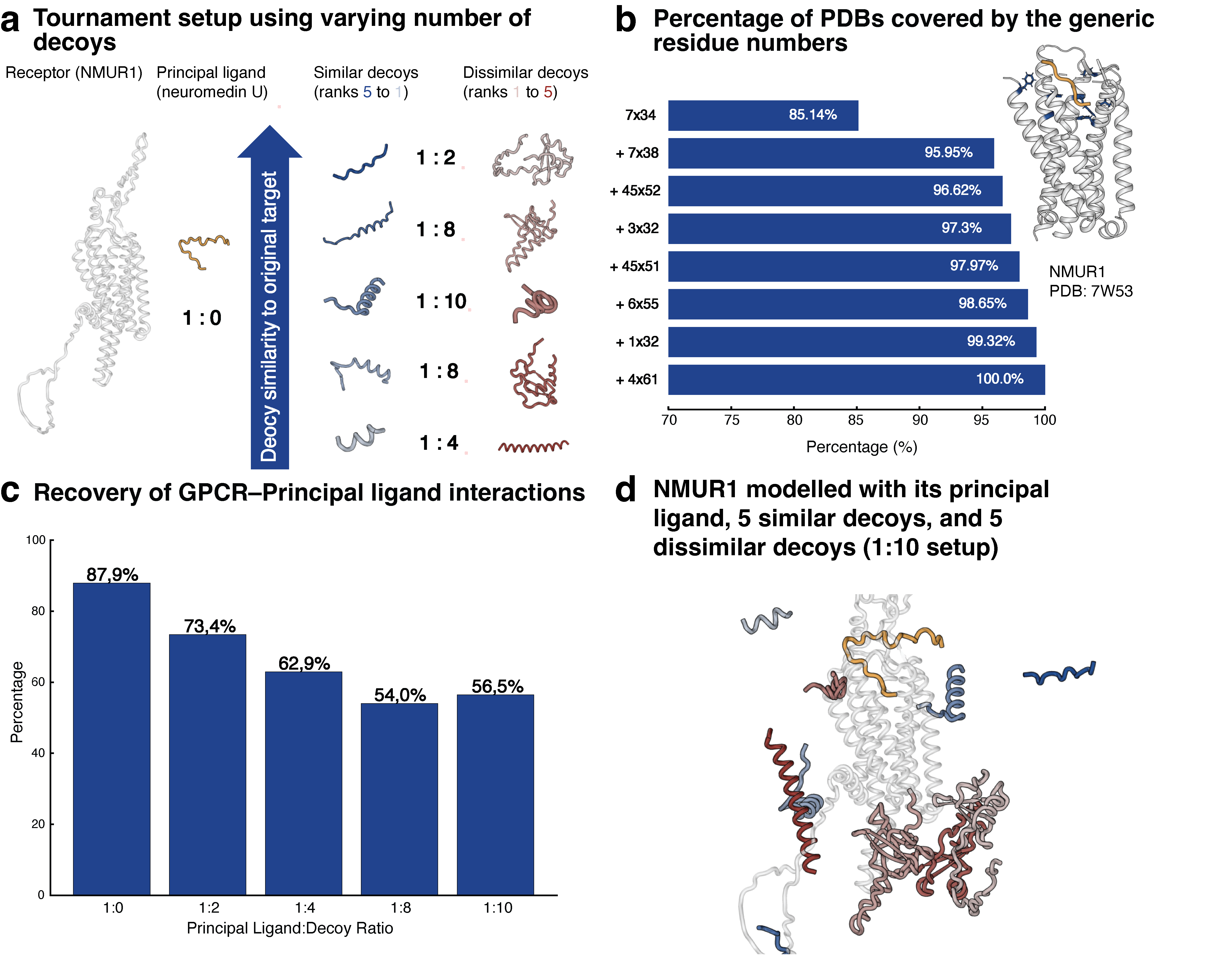
**

**Supplementary Figure 5: Tournament benchmark using an increasing number of decoy peptides.** **a**, the tournament benchmark was done by modelling GPCRs with their principal ligands and an increasing number of decoys. The labels represent the principal peptide ligand:decoy ratios in the models. **b**, eight unique GRNs defining the orthosteric binding pocket, visualized on neuromedin U receptor 1 (NMUR1). **c**, the produced complex models by AF2 with varying numbers of decoys were analyzed by calculating the frequency of principal ligands interacting with the orthosteric binding pocket. Unsurprisingly, when no decoys were included in the model, the recovery is the highest, and the performance declines as more decoys are modelled along the GPCR and its principal ligand. **d**, an example of the produced complex prediction for 1:10 setup using NMUR1 with neuromedin U (GtP ID: 1,470) and five similar and dissimilar decoys.

**
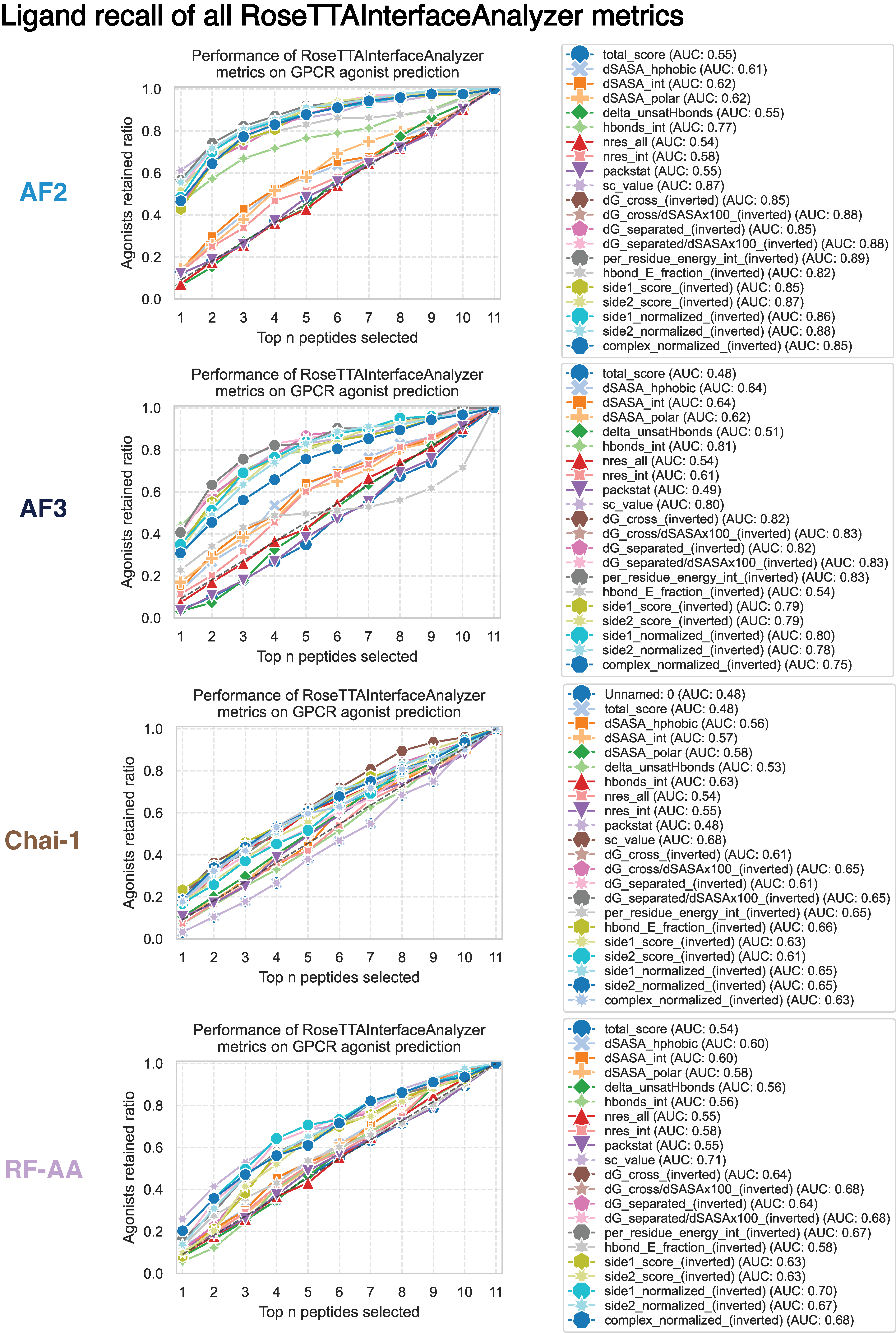
**

**Supplementary Figure 6: Performance as a peptide classifier using RoseTTAInterfaceAnalyzer metrics for AF2, AF3, Chai-1, and RF-AA.** The curves show the number of true agonists retained, when selecting the top n peptides based on the re-scoring metric from RoseTTAinterface analyzer. A higher AUC means the metric is better at distinguishing real agonists from decoys.

**
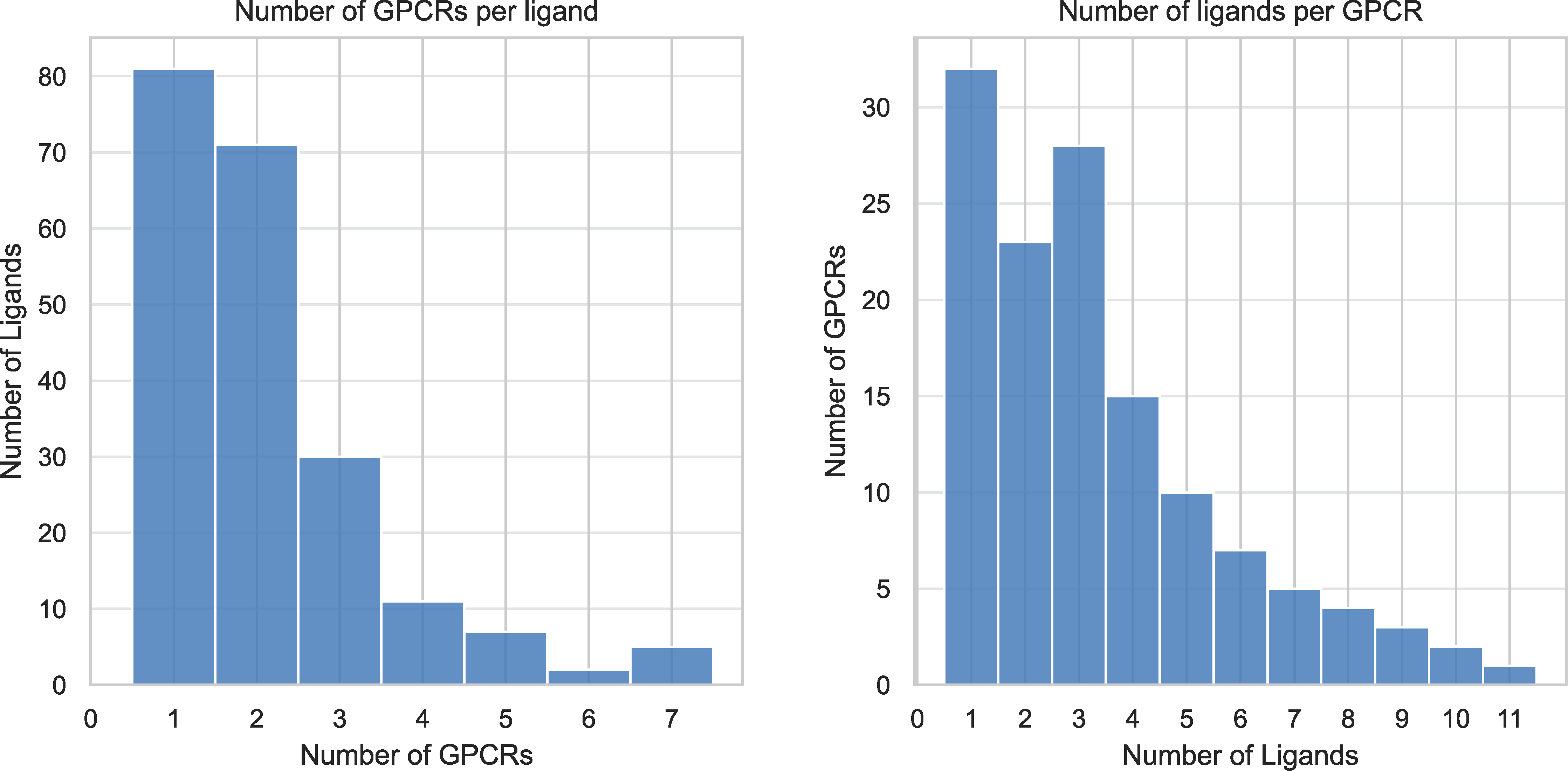
**

**Supplementary Figure 7:** The figure shows how many endogenous peptide ligands are paired with unique GPCRs (*left*) and conversely, how many endogenous peptide ligands each GPCR (*right*) has in the classifier benchmark dataset as by IUPHAR.


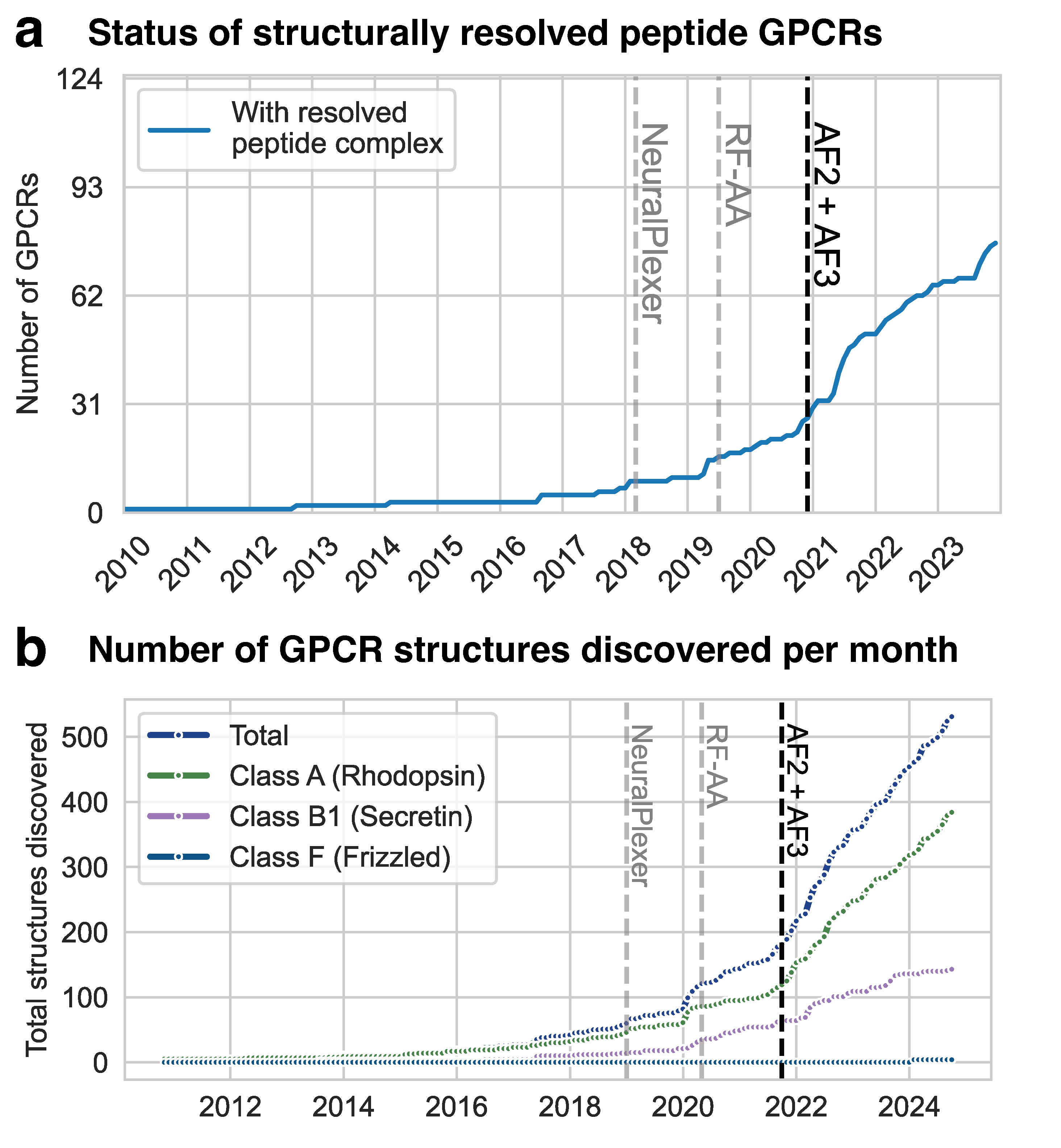


**Supplementary Figure 8: Number of resolved GPCR structures over time.** **a,** Nearly 80 of the 124 human peptide GPCRs have been structurally characterized in complex with a peptide ligand (GPCRdb [1], 20^th^ of November 2024). **b,** The amount of experimentally determined structures discovered over time (GPCRdb [1], 20^th^ of November 2024). Dashed lines indicate model training cut-offs for NeuralPLexer, RF-AA, and AF2/3.

**
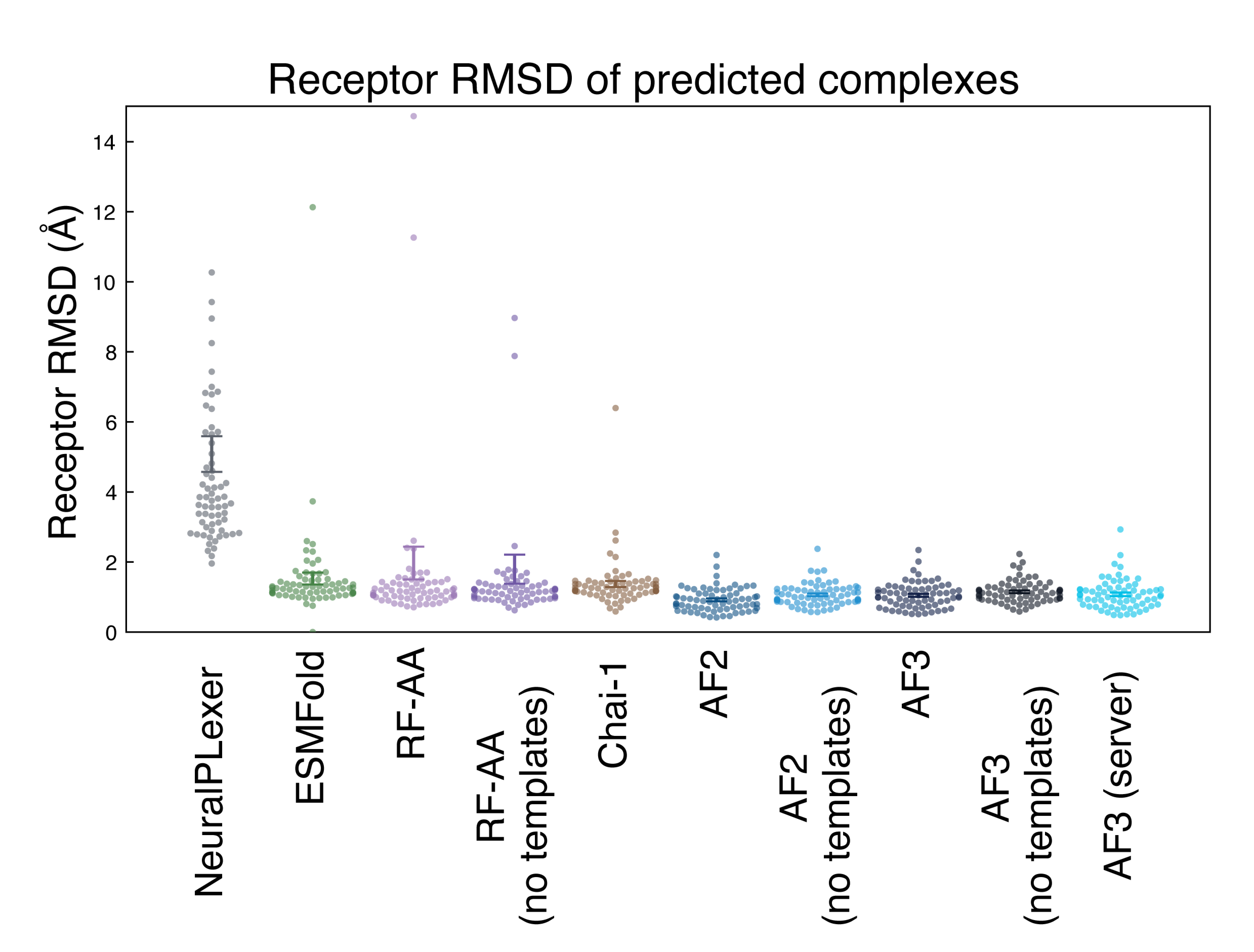
**

**Supplementary Figure 9: Receptor backbone RMSD of structural benchmark predictions.** Although many of the models failed to model the GPCR–peptide interaction accurately, namely ESMFold and RF-AA, these same models produce highly accurate predictions of the GPCR backbone. The standard error of the mean is shown for all models.

**
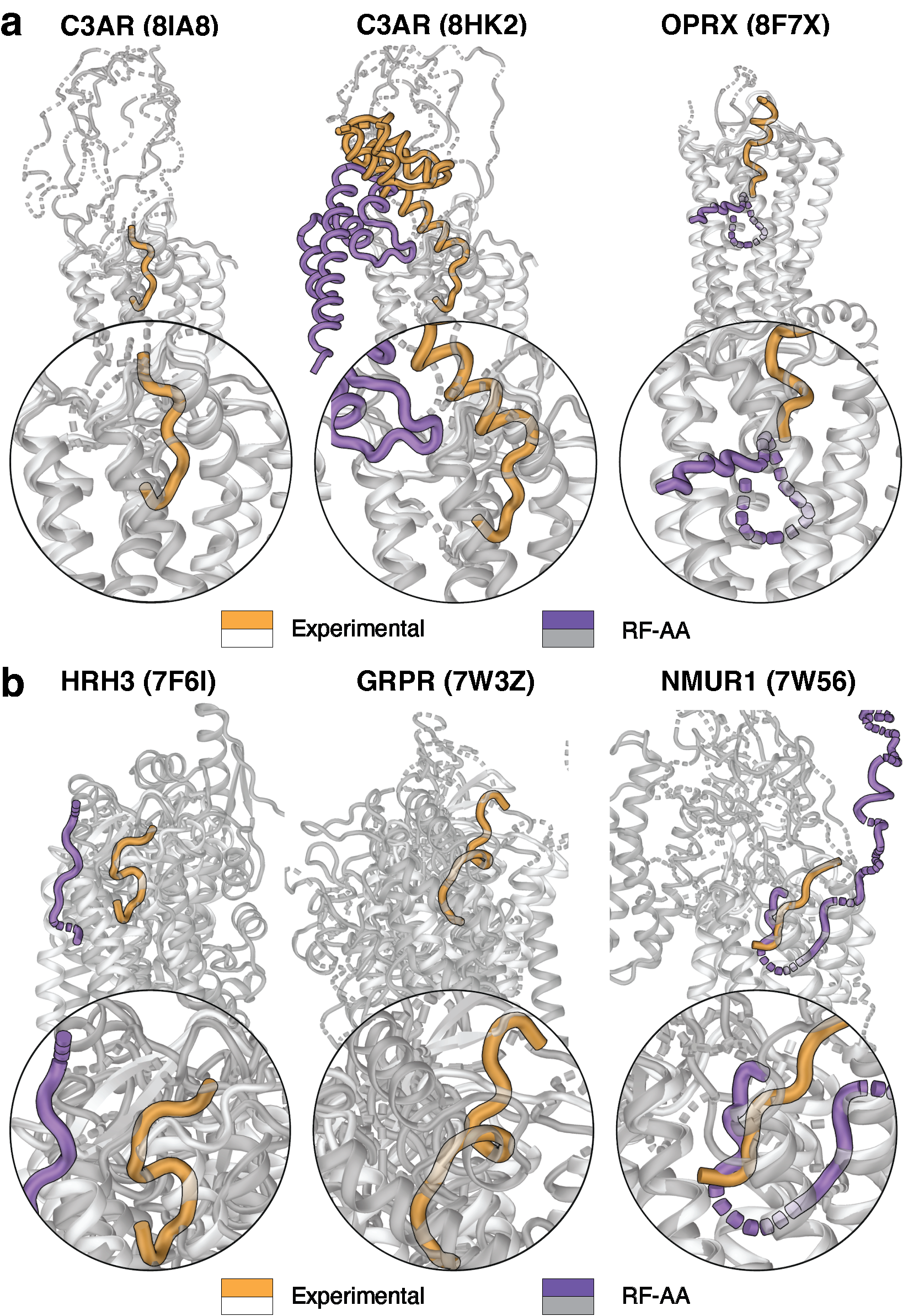
**

**Supplementary Figure 10: Failed RF-AA predictions.** Examples of RF-AA failing to dock the peptide correctly, due to receptor sequence being folded into the binding pocket. The predicted GPCR–peptide complex is shown in grey and purple while the experimental complex is visualized in white and orange. **a,** visualizes cases in which the receptor is modelled highly similarly to the experimental model, but with the extracellular loop folding into the binding pocket. **b,** cases in which large extracellular loops are modelled incorrectly, producing unreliable GPCR backbone conformations.


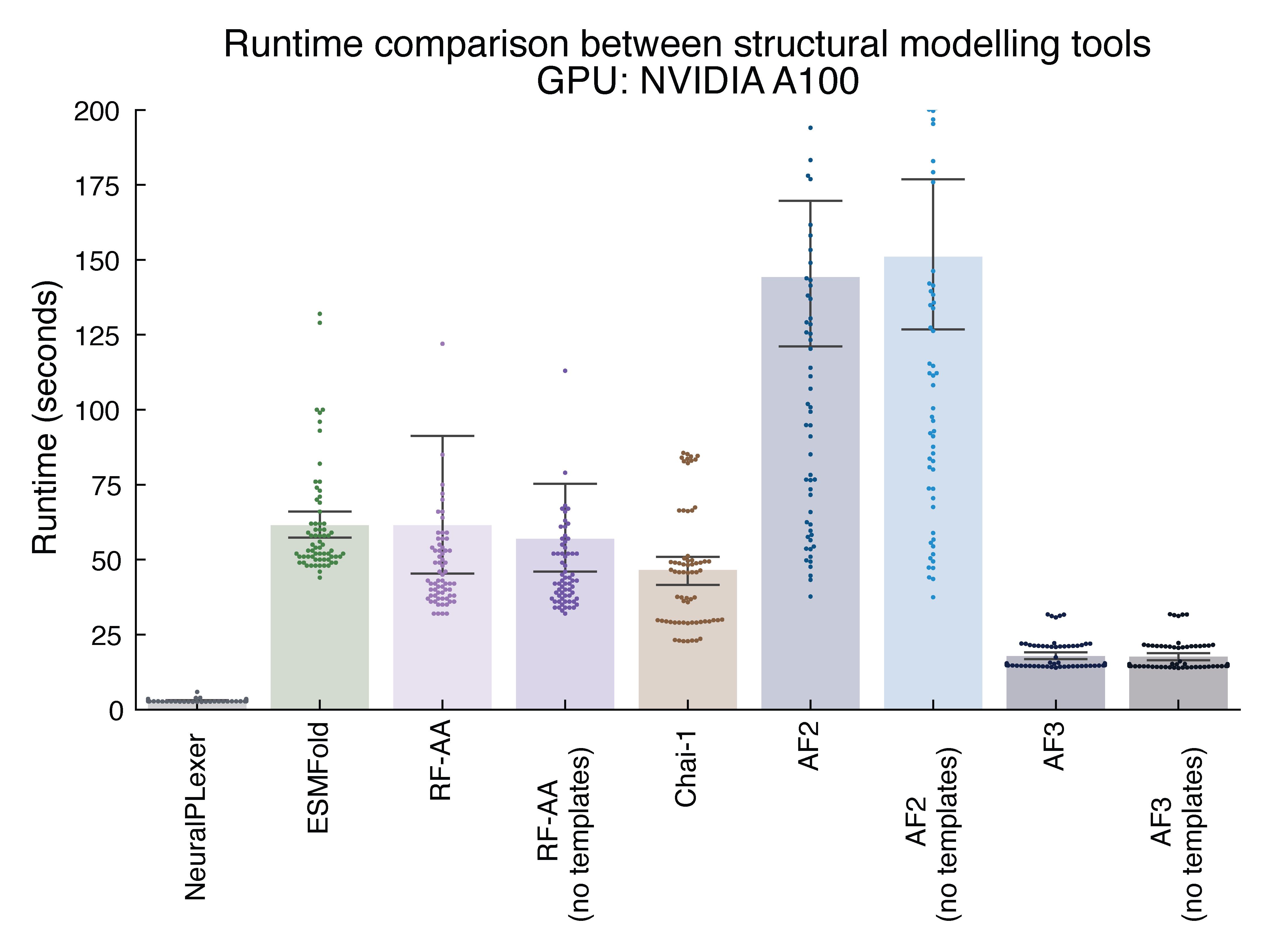


**Supplementary Figure 11: Runtime comparison between structural modelling tools.** We compared the required runtimes to generate structural predictions of GPCR–peptide complexes. All tools were run on NVIDIA A100 GPUs. As the visualized runtimes show the time required to generate a single prediction, it should be noted that the number of generated structures varies; ESMFold and RF-AA generate a single prediction, AF3 and Chai-1 generate five predictions, NeuralPLexer outputs 16 conformations, and AF2 generates 25 structures by default in total. AF2 is therefore not only the slowest at generating a singular prediction but its total runtime is also considerably longer compared to all other included modelling tools. The runtimes shown here also exclude the input MSA preparation, which is required for RF-AA, AF2, and AF3, while ESMFold, Chai-1 and NeuralPLexer do not require MSAs.


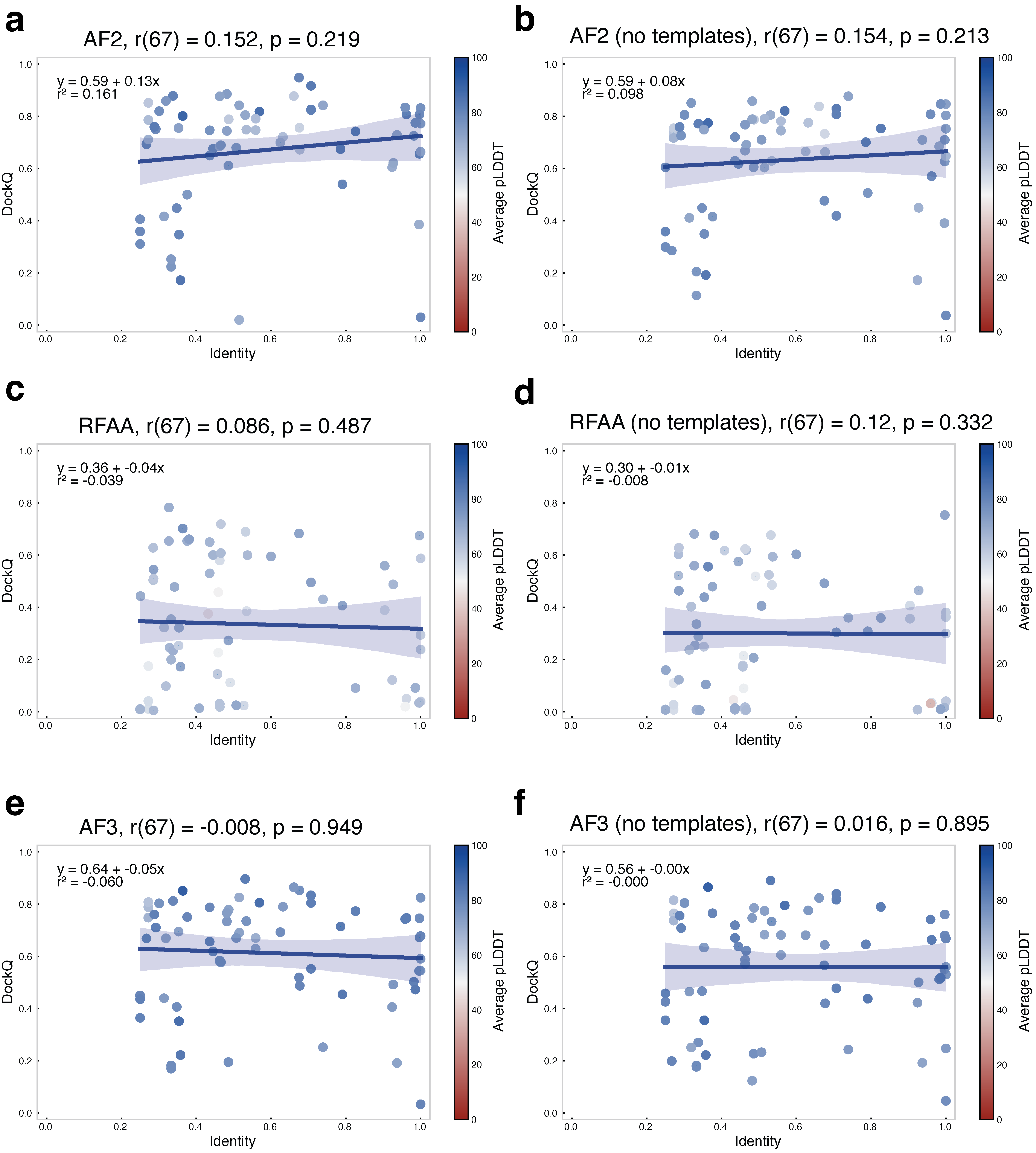


**Supplementary Figure 12: Closest training structure sequence identity vs DockQ score.** To see whether the DockQ score was significantly correlated with closest training sequence identity, we searched the training structure databases of RF-AA (**a/b**), AF2 (**c/d**), and AF3 (**e/f**), for similar sequences using MMseqs2 [2]. DockQ score is not significantly correlated with the closest training structure sequence identity, suggesting that all models generalize well to novel inputs. This suggests that DockQ score performance is not explained by the presence of a highly similar training structure. Furthermore, uniformly confident predictions, as measured by averaged pLDDT score, were produced for all inputs, albeit varying levels of sequence identity to the closest training structures.

**REFERENCES**

1. Pandy-Szekeres G, Caroli J, Mamyrbekov A et al. GPCRdb in 2023: state-specific structure models using AlphaFold2 and new ligand resources, Nucleic Acids Res 2023;51:D395-D402.

2. Steinegger M, Söding J. MMseqs2 enables sensitive protein sequence searching for the analysis of massive data sets, Nature biotechnology 2017;35:1026-1028.
