# Supplementary material for: Deep learning in GPCR drug discovery: benchmarking the path to accurate peptide binding
Source: Brief Bioinform. 2025 Apr 26;26(2):bbaf186. doi: 10.1093/bib/bbaf186 (PMC12031724; doi:10.1093/bib/bbaf186)
Supplement: Supplementary_Methods_bbaf186 [file supplementary_methods_bbaf186.docx]

**SUPPLEMENTARY METHODS**

**Competitive tournament approach**

To model GPCR–peptide interactions in the competitive tournament setup, AF2 was run without structural templates, using identical settings as described earlier for the classifier benchmark. First, all 124 GPCRs included in the classifier dataset were modelled together with their principal ligands. Next, the most and least similar decoys from the classifier dataset are added to the models. In subsequent models, more decoys were added based on decoy peptide similarity, as defined earlier. In this way, we produced five unique predictions for each GPCR, so that the principal ligand-to-decoy ratios in these models were 1:0, 1:2, 1:4, 1:8, and 1:10, meaning that in these setups, each GPCR was modelled with one, three, five, nine, and 11 peptides.

To define the orthosteric binding pocket across class A and B1 GPCRs, we retrieved available structural models of GPCR–peptide interactions from the GPCRdb [1]. In total, 148 PDB structures were retrieved, covering 68 unique GPCRs belonging to classes A (54) and B1 (14). The models were retrieved in October 2024 using the GPCRdb API. Using the annotated GPCR–peptide interactions on the GPCRdb, we parsed all GPCR–peptide interactions involving a GRN. Because GRNs can be mapped between different GPCRs belonging to classes A and B1, we chose to use the GRN positions of class A as the reference for all GPCRs. We then chose the most frequently appearing GRNs that were involved in GPCR–peptide interactions by finding the minimum number of GRNs required to cover all GPCR–peptide interactions annotated in the 148 experimental models. These chosen GRNs were then used to define the orthosteric binding pocket using a distance cut-off of 6.6 Å to find predicted complexes in which there was an interaction between a GPCR GRN and any atom belonging to the peptide. The distance cut-off was chosen to be identical to that used in the GPCRdb source code (<https://github.com/protwis/protwis>) for detecting interacting residues [1]. If the minimum distance between GPCR GRN and any peptide atom was less than 6.6 Å, we considered the peptide to be correctly placed in the orthosteric binding pocket.

**Correlation analysis between DockQ score and sequence identity of the closest training sequence**

To determine whether the prediction accuracy of AF2, AF3, and RF-AA was driven by the presence of similar training structures, we used MMseqs2 [2] to search the training databases of AF and RF-AA for similar training structures for each GPCRs, based on the GPCR sequence. The databases for the MMseqs2 search were built using the mmseqs createdb, mmseqs createtaxdb, and mmseqs createindex commands, and the search was performed using the following command:

mmseqs easy-search --alignment-mode 3 -s 7.0 $INPUT_FASTA $INPUT_DB $OUTPUT_DIR/$BASENAME.m8 tmp

The sequence identity of the closest training structure was correlated with the DockQ score using Spearman’s rank correlation from scipy.stats.

**REFERENCES**

1. Pandy-Szekeres G, Caroli J, Mamyrbekov A et al. GPCRdb in 2023: state-specific structure models using AlphaFold2 and new ligand resources, Nucleic Acids Res 2023;51:D395-D402.

2. Steinegger M, Söding J. MMseqs2 enables sensitive protein sequence searching for the analysis of massive data sets, Nature biotechnology 2017;35:1026-1028.
