# Supplementary material for: Deep learning in GPCR drug discovery: benchmarking the path to accurate peptide binding
Source: Brief Bioinform. 2025 Apr 26;26(2):bbaf186. doi: 10.1093/bib/bbaf186 (PMC12031724; doi:10.1093/bib/bbaf186)
Supplement: Supplementary_Results_bbaf186 [file supplementary_results_bbaf186.docx]

**SUPPLEMENTARY RESULTS**

**A competitive tournament approach with AF2** **accelerates peptide discrimination**

Since AF2 performed the best overall, we next evaluated its capability to simultaneously model multiple peptides in complex with a receptor, focusing on its accuracy in positioning the principal ligand in the orthosteric binding pocket while excluding decoys. In this approach, each receptor is modelled with its cognate peptide ligand and an increasing numbers of decoys. This resulted in GPCR–peptide models with principal ligand:decoy ratios of 1:0, 1:2, 1:4, 1:8, and 1:10. For each modelling task, 124 GPCR–peptide models were generated, resulting in 620 predictions.

To assess sensible binding modes, we proxied the orthosteric binding pocket from structural information from 148 experimental GPCR–peptide models from the GPCRdb [1]. We identified 109 unique residue positions among class A and B1 receptors. Interactions with eight unique residues were required to recapitulate the experimental GPCR–peptide interactions defining the orthosteric peptide-binding pocket (**Supplementary Fig. 5b**). The chosen GRNs were mainly distributed across the transmembrane domains of the GPCRs (**Supplementary Fig. 3**). AF2 correctly placed the principal ligand in the binding pocket in 87,9% (109/124) of the models when the complex only included the GPCR and principal ligand. In the remaining 15 models, the peptide was most often placed on the intracellular side of the GPCR. In a competitive setup, including more similar and dissimilar peptides reduced the percentage of principal ligand retention to 73,4% (1:2), 62,9% (1:4), 54,0% (1:8), and 56,5% (1:10) (**Supplementary Fig. 5c**).

We wanted to determine whether using a tournament setup would result in a significantly faster interaction screening time. Hence, we compared the AF2 runtimes of all one-to-many GPCR–peptide complexes with those from one-to-one complex predictions. The tournament setup was significantly faster for each principal ligand-to-decoy ratio (Wilcoxon signed-rank test, p < .001, see **Supplementary Table 4**), and the relative speedup achieved by modelling a receptor with more peptides at once increased when more decoy peptides were included in the model (**Supplementary Fig. 4**).

**REFERENCES**

1. Pandy-Szekeres G, Caroli J, Mamyrbekov A et al. GPCRdb in 2023: state-specific structure models using AlphaFold2 and new ligand resources, Nucleic Acids Res 2023;51:D395-D402.
